# Adaptation of faba bean cultivars across Norwegian environments: identification of elite material and definition of optimal breeding and cultivar testing procedures

**DOI:** 10.3389/fpls.2026.1936638

**Published:** 2026-09-08

**Authors:** Stefano Zanotto, Jon Arne Dieseth, Wendy Waalen, Paolo Annicchiarico, Anne Kjersti Uhlen

**Affiliations:** 1 Department of Plant Science, Faculty of Biosciences, Norwegian University of Life Sciences (NMBU), Ås, Norway; 2 Council for Agricultural Research and Economics (CREA), Research Centre for Animal Production and Aquaculture, Lodi, Italy; 3 Graminor AS, Hamar, Norway; 4 Division of Food Production and Society, Norwegian Institute for Bioeconomy Research (NIBIO), Ås, Norway

**Keywords:** AMMI, cultivar adaptation, factorial regression, genotype × environment interaction, multi-environment trials, plant breeding, *Vicia faba* L.

## Abstract

**Background:**

Faba bean cultivation in northern Europe is challenged by high variability in yield performance due to strong genotype × environment interaction (GEI). Appropriate GEI modeling and interpretation is essential for both cultivar recommendation and breeding for Nordic conditions.

**Methods:**

Grain yield data from fifteen spring faba bean cultivars evaluated in eleven Norwegian environments during four growing seasons (2022–2025) were analyzed using the Additive Main Effects and Multiplicative Interaction (AMMI) model. Climatic drivers of GEI were investigated using factorial regression, while genome-wide SNP markers were used to assess the relationship between molecular diversity and adaptive differentiation.

**Results:**

Environment, genotype, and GEI significantly influenced grain yield. The AMMI model with three GEI principal components (AMMI3) represented the biologically meaningful GEI signal and explained 83.4% of the GEI variation. The predictive accuracy of this model closely approached that of BLUP and GBLUP. Factorial regression showed that maximum and mean September temperatures explained 38.5% of the GEI variation, indicating that late-season thermal conditions are the principal environmental drivers of cultivar adaptation. We identified three top-yielding genotypes (Ketu, Birgit, and Vire) that can be object of site-specific recommendation. Two sub-regions emerged as possibly distinct breeding targets, namely, a major one, and a smaller one featuring cooler late-cycle temperatures and specific adaptation of very early material. A significant Mantel correlation between genomic and adaptive dissimilarity (r = 0.40, P = 0.029) suggested that differences in environmental adaptation have a measurable genetic basis.

**Conclusions:**

This study identified elite cultivars for recommendation and generated crucial information for future crop breeding and variety testing in Norway. Breeding for wide adaptation across climatically diversified environments is justified by the modest size of the smaller sub-region and its expected decreasing importance due to climate change. The relationship between genomic and adaptive dissimilarity, if confirmed for a larger genotype set, could be exploited for a preliminary screening of regional breeding material and novel plant introductions.

## 1 Introduction

Faba bean (*Vicia faba* L.) has become one of the most promising grain legumes for temperate agriculture because of its high seed protein content (Khazaei and Vandenberg, 2020), biological nitrogen fixation (Klippenstein et al., 2021), and contribution to more sustainable cropping systems (Köpke and Nemecek, 2010). The increasing demand for locally produced plant protein in Europe (de Visser et al., 2014) has renewed the interest of expanding faba bean cultivation into regions where the crop historically occupied only a limited area. In the Nordic countries, faba bean represents the most valuable alternative to imported soya bean products. In Norway, in particular, there is large scope in increasing faba bean production (Abrahamsen et al., 2018), also considering the ongoing climate change which is progressively extending the growing season and the suitability of northern environments for agricultural production (Ergon et al., 2018). Nevertheless, the successful expansion of faba bean cultivation requires cultivars that combine high yield potential with reliable adaptation to the considerable environmental variability typical of Nordic climates.

One of the major challenges in cultivar evaluation is the presence of genotype × environment interaction (GEI). GEI complicates cultivar recommendation and breeding activities because genotypes selected on one environment may fail to perform consistently under different environmental conditions. A thorough understanding of GEI patterns provides an opportunity to improve productivity by matching cultivars to the environments for which they are best adapted and by defining optimal strategies for future breeding activities (Annicchiarico, 2002). Hence, the analysis of multi-environment trials (METs) represents a fundamental component of modern cultivar testing and breeding programs. In particular, the modeling of GEI effects in METs can help understand the adaptability and stability of genotypes across a wide range of environments and its implications for regional breeding. Such an approach for faba bean in southern Europe highlighted the presence of two major sub-regions with different climate (subcontinental or truly Mediterranean) that should be object of specific breeding, owing to contrasting and largely incompatible earliness and stress tolerance of plant types specifically adapted to each of them (Annicchiarico and Iannucci, 2008).

Numerous statistical approaches have been developed to investigate GEI. Mixed-model methodologies based on Best Linear Unbiased Prediction (BLUP) have become increasingly popular for the analysis of METs because of their flexibility and predictive performance, and have been applied in several species, including faba bean (Gela et al., 2023; Olivoto et al., 2019). Although these approaches provide powerful prediction tools, if applied alone, they generally offer limited biological interpretation of the GEI structure itself. Conversely, the Additive Main Effects and Multiplicative Interaction (AMMI) model (Gauch, 1992) has become one of the most widely used approaches for analyzing MET data. By combining analysis of variance for genotype and environment main effects with principal component analysis of the GEI matrix, AMMI separates meaningful interaction patterns from experimental noise while providing an intuitive graphical interpretation of cultivar adaptation (Gauch et al., 2008). Furthermore, AMMI can be used to delineate mega-environments for specific cultivar recommendations and to identify broadly or specifically adapted cultivars, as well as for location classification aimed to identify possible sub-regions object of specific breeding (Annicchiarico, 2002).

Environmental covariates offer an additional opportunity to improve the biological interpretation of GEI (van Eeuwijk et al., 1996). Whereas AMMI effectively summarizes interaction patterns, approaches such as factorial regression (Denis, 1988) of GEI effects with environmental covariates relates genotype responses directly to measurable environmental variables, thereby investigating likely agroclimatic factors responsible for differential cultivar performance. Such complementary analyses can provide valuable insights into the physiological basis of adaptation and performance and support the definition of breeding targets under future climatic conditions (Annicchiarico, 2002).

In parallel with statistical models based on phenotypic responses across environments, genomic prediction approaches such as genomic best linear unbiased prediction (GBLUP) have become widely adopted in plant breeding. By including a genomic relationship matrix derived from genome-wide molecular markers, GBLUP exploits the genetic relatedness among individuals to predict breeding values and has demonstrated high predictive ability for complex quantitative traits across several crop species (Meher et al., 2022).

This study aimed to strengthen the cultivation of faba bean in Norway and other Nordic regions by generating useful information for cultivar targeting to specific environments and for the definition of optimal breeding strategies. Its specific objectives were (i) to model and interpret GEI patterns for grain yield of fifteen spring-sown faba bean cultivars grown in eleven Norwegian environments using AMMI and factorial regression models; (ii) to compare the predictive performance of AMMI with a conventional BLUP model and with a GBLUP model including genomic data; (iii) to identify top-performing cultivars which can be recommended for cultivation in different Norwegian mega-environments; (iv) to verify the possibility to breed for wide adaptation to Norwegian environments or for specific adaptation to distinct sub-regions; and (v) to use genomic data to investigate the relationship between molecular diversity and adaptive differentiation using genome-wide SNP markers.

## 2 Materials and methods

### 2.1 Plant material and field trials

The yield data used in this work were produced in field trials for variety testing of faba bean in three locations of Norway during the seasons 2022-2025. The selection of varieties was done through yearly contact with breeding companies in northern and central Europe, which provided cultivars of spring faba bean that were assumed to be adapted to Nordic climates. A set of fifteen cultivars were included in field trials at all locations and years and were selected for the present study. **Supplementary Table 1 (S1)** gives an overview of the varieties and their origin. The three locations were Vollebekk (research farm at Norwegian University of Life Science, Ås, 59°39′ N, 10°45′ E), Bjørke or Staur (research farms of Graminor AS, Hamar, 60°80′ N, 11°20′ E or 60°73′ N, 11°11′ E, respectively), and Apelsvoll (research farm at the Norwegian Institute of Bioeconomy Research, Toten, 60°70′ N, 10°87′ E). Since the research farms Bjørke and Staur are located close to each other with similar weather and soil conditions, we will refer hereafter only to Bjørke to indicate this location. All locations are within the main cereal producing region in southeast of Norway. Among these, Apelsvoll is representative for growth conditions of slightly cooler temperatures and with shorter duration of the growth season, whereas Vollebekk is representative for growth conditions of slightly warmer temperatures, early sowing time, and longer growth season. In total, data from eleven single field trials were collected, representing eleven environments (hereafter referred to as location-year combinations). An overview of the daily mean temperature and precipitation at the eleven environments is provided in **Supplementary Figure 1 (S2)**. The field trials were laid out in a randomized complete block design with four replicates. At seven of the eleven environments two replicates were treated with fungicide (mainly against chocolate spot) while the two other replicates were untreated. For the remaining four environments, fungicide was applied to all four replicates. Details of management practices applied to each field trial are reported in **Supplementary Table 2 (S3)**. Seed rates were set to 60 viable seeds/m^2^ for late varieties and 80 seeds/m^2^ for early, small-seed (minor type) varieties. The trials were sown when the soil reached optimal moisture content in the spring. Plot size varied slightly at the different locations, from 9,6 m^2^ at Bjørke to 12,8 m^2^ at Apelsvoll. Weeds were controlled by using the herbicide Basagran (Bentazon, 870 g/kg). The field trials were carried out on fertile soil of optimal pH. No fertilization nor Rhizobium inoculation was applied, but a PK fertilizer was applied to secure optimal plant growth (accurate dosage at each environment is provided in **Supplementary Table 2, (S3)**). The field trials were harvested using a plot combine harvester. A seed lot per plot was collected for moisture assessment, expressing grain yield in t/ha at 15% moisture.

### 2.2 Genomic data

Genotypic data for 14 faba bean genotypes (genotype Sampo was discarded because of too many missing data) were obtained using the targeted Genotyping-by-Sequencing Single Primer Enrichment Technology (GBS-SPET) platform (Scaglione et al., 2019) provided by IGA Technology Services (Udine, Italy). Marker quality filtering, imputation, and construction of the final SNP dataset were performed following the same bioinformatic pipeline described in Zanotto et al. (2025). Briefly, SNPs with more than 20% missing values or a minor allele frequency (MAF) below 0.01 were removed. Remaining missing genotype calls were imputed by replacing each missing value with the mean allele frequency of the corresponding SNP across all genotypes. The resulting filtered marker matrix was used both for calculation for the analyses described in sections 2.3.3 and 2.4.

### 2.3 Statistical analyses

For practical reasons related to a more appropriate description of the following statistical analyses, hereafter we will refer to cultivars as genotypes.

#### 2.3.1 Phenotypic adjustment

Grain yield values were adjusted for fungicide treatment in environments where both treated and untreated plots were present. Since the investigation of fungicide response was beyond the scope of this study, this adjustment was performed to harmonize environments and obtain a balanced number of replications for subsequent multi-environment analyses. Within each environment, the fungicide effect was estimated using the following linear model:


$$Y_{ij}=\mu +F_{i}+G_{j}+\epsilon_{ij}$$

where *Y_ij_* is the observed grain yield, *m* is the overall mean, *F_i_* is the fixed effect of fungicide treatment *i*, *G_j_* is the effect of genotype *j*, and *e_ij_* is the residual error.

To verify the assumption of an additive fungicide effect, a model including the fungicide × genotype interaction was also fitted separately for each environment. No consistent interaction was detected across environments, with a significant interaction observed only in one environment (Bj24).

The estimated fungicide effect was then added to untreated plots, thereby expressing all observations on a fungicide-treated scale. Environments in which only one fungicide treatment level was present were not adjusted, as all plots had received fungicide, reflecting standard agronomic practice for faba bean cultivation in Norway.

#### 2.3.2 Additive main effects and multiplicative interaction analysis

Genotype × environment interaction (GEI) for grain yield was investigated using the Additive Main Effects and Multiplicative Interaction (AMMI) model implemented in the R package metan (Olivoto and Lúcio, 2020). The AMMI approach first partitions the total variation into genotype (G), environment (E), and genotype × environment interaction (GEI) effects through analysis of variance (ANOVA) and subsequently decomposes the interaction matrix using principal component analysis (PCA). Model diagnosis and selection were performed using three complementary approaches. Firstly, the significance of GEI principal component axes (IPCAs) was assessed using the *FR* ratio test (Cornelius et al., 1992) as recommended by Piepho (1994). Secondly, the proportion of GEI variation explained by each IPCA and the cumulative variance explained were used to describe the interaction structure using the signal-to-noise criterion proposed by Gauch (2013), by which the interaction noise component (*GEN*) was estimated as:


GEN=df_GEI×MS_Error

where *df_GEI* is the degrees of freedom associated with the genotype × environment interaction and *MS_Error* is the residual mean square from the ANOVA.

The interaction signal (*GES*) was then estimated as:


GES=SS_GEI−GEN

where *SS_GEI* is the sum of squares of the GEI. The number of IPCAs retained was chosen such that the cumulative interaction sum of squares explained by the retained IPCAs approximated the estimated interaction signal (*GES*).

Thirdly, predictive accuracy of the AMMI-family models was further assessed by cross-validation using the cv_ammif() function in metan. Cross-validation was conducted using 200 resampling iterations, and model performance was assessed through the root mean square predictive difference (RMSPD). Final model selection was based on agreement between predictive accuracy and the signal-to-noise criterion while favoring the most parsimonious model.

#### 2.3.3 Mixed-model and genomic prediction analyses

To compare the predictive performance of AMMI with mixed-model approaches, phenotypic BLUP and genomic BLUP (GBLUP) models were fitted. BLUP and GBLUP analyses were conducted with the sommer package (Covarrubias-Pazaran, 2016) using the mixed model:


y=Xβ+Zu+ZGEuGE+e

where y is the vector of phenotypic observations, *X* is the design matrix for the fixed environmental effects where y is the vector of phenotypic observations, *X* is the design matrix for the fixed environmental effects where y is the vector of phenotypic observations, *X* is the design matrix for the fixed environmental effects *β*, *Z* is the incidence matrix linking observations to genotype effects *u, ZGE* is the incidence matrix linking observations to genotype × environment effects *uGE*, and e is the vector of residual errors.

For the BLUP model:


$$u∼N(0,I\sigma^{2}g)$$

For the GBLUP model:


$$u∼N(0,G\sigma^{2}g)$$

where *I* is the identity matrix and *G* is the genomic relationship matrix calculated from genome-wide SNP markers, used to model the main genotypic effect, whereas the genotype × environment interaction was included as a conventional random effect without a genomic covariance structure. The genomic dataset and marker processing are described in section 2.2. A custom cross-validation pipeline reproducing the one available in the metan package, with resampling strategy (200 iterations) was implemented for the models fitted with the sommer package, and predictive ability was quantified using RMSPD to enable direct comparison among AMMI, BLUP and GBLUP models.

#### 2.3.4 Environmental characterization and factorial regression analysis

Monthly climatic variables were compiled for each location-year combination to characterize environmental variation among environments. For each month, average daily mean, minimum and maximum air temperature and total precipitation were obtained for the growing period from May to September common across environments, resulting in 20 environmental covariates. Principal component analysis (PCA) was performed on the standardized environmental variables to summarize environmental similarity among trial environments and identify the main climatic gradients. To identify climatic variables underlying GEI, factorial regression (FR) was performed as described in Annicchiarico (2002). FR analysis was conducted on the genotype × environment interaction effects matrix after removal of genotype and environment main effects according to the model:


$$GL_{ij}=\alpha_{i}+\sum \beta_{in}V_{jn}+d_{ij}$$

where *GL_ij_* is the interaction effect of genotype *i* in environment *j*, *α_i_* is the genotype-specific intercept, *β_in_* is the sensitivity of genotype *i* to environmental covariate *n*, *V_jn_* is the value of environmental covariate *n* in environment *j*, and *d_ij_* is the residual deviation. Each environmental covariate was first evaluated individually by fitting a one-covariate factorial regression model. The significance of the genotype × covariate term was assessed with F-test statistic. Candidate variables were then entered into a forward-selection procedure, retaining additional genotype × covariate terms only when they significantly improved model fit according to the Akaike’s Information Criterion (AIC). Because variables were added sequentially, a covariate was retained only if it provided additional explanatory power, thereby limiting the inclusion of redundant environmental predictors. For the final model, the proportion of GEI explained by each retained environmental variable was calculated as explained in Annicchiarico (2002) multiplying the model R² by the total GEI sum of squares obtained from the AMMI analysis, allowing the contribution of each covariate to be expressed as a percentage of the total GEI.

Finally, the expected yield responses to environmental change was expressed in terms of nominal yields (*N_ij_*) calculated according to:


$$N_{ij}=m_{i}+\alpha_{i}+\sum \beta_{in}\;V_{jn}$$

where *N_ij_* is the nominal yield of genotype *i*, *m_i_* is its mean yield across environments, *α_i_* is the genotype intercept, and, *β_in_* is the sensitivity of genotype *i* to environmental covariate *n*, *V_jn_* is the value of environmental covariate *n.* in environment *j*. Nominal yields were calculated at the minimum and maximum observed values of the significant environmental covariate(s) to visualize genotypes response.

### 2.4 Integration of genomic data to explore patterns of adaptation

The genetic structure was investigated using Discriminant Analysis of Principal Components (DAPC) implemented in the adegenet package (Jombart and Ahmed, 2011). To investigate the relationship between molecular diversity and adaptive dissimilarity, a genomic dissimilarity matrix was calculated as Euclidean distances among genotypes based on the filtered SNP marker matrix. Adaptive dissimilarity was obtained from the genotype dissimilarity matrix generated by the Pattern Analysis of environment-standardized yield data (DeLacy et al., 1996), which simultaneously accounts for differences in mean yield and GEI patterns. The correspondence between genomic and adaptive dissimilarity matrices was assessed using the Mantel test (Mantel, 1967) with 9,999 permutations.

## 3 Results

### 3.1 AMMI analysis of genotype × environment interaction

#### 3.1.1 Sources of variation and AMMI model selection

The AMMI analysis revealed highly significant effects of environment, genotype and genotype × environment interaction (GEI) for grain yield (**Table 1**). The *FR*-ratio test indicated significance of the first three IPCAs. The signal-to-noise method suggested by Gauch (2013) for model diagnosis confirmed AMMI3 as the model with best balance between model complexity and biological signal. Cross-validation further supported this conclusion (**Supplementary Figure 2, (S4)**), as the improvement predictive accuracy reached a plateau after the third interaction principal component.

**Table 1:** Analysis of variance for additive main effects and multiplicative interaction (AMMI) analysis for grain yield of 15 faba bean genotypes evaluated at 11 environments during 2022–2025 cropping seasons.

Source	df	SS	MS	F	P value	Variation explained	GEI (%)	Cum. GEI (%)
Environment	10	11602530	1160253	164.592	<0.001	67.86		
Rep (Environment)	33	232625	7049	2.801	<0.001	1.36		
Genotype	14	1766967	126212	50.138	<0.001	10.33		
GEI	140	2332244	16659	6.617	<0.001	13.64		
IPCA1	23	1087656	47289	4.226	<0.001		46.6	46.6
IPCA2	21	477158	22722	3.176	<0.001		20.5	67.1
IPCA3	19	381250	20066	1.992	0.0078		16.3	83.4
IPCA4	17	165434	9731	1.462*	0.104		7.1	90.5
Pooled experimental error	462	1162984	2517			6.80		

Significance levels for the interaction principal components were tested with the *FR* ratio test.

*Only the first 6 IPCAs are reported which account for 96.9% of GEI.

#### 3.1.2 Structure of genotype × environment interaction

**Figure 1:**
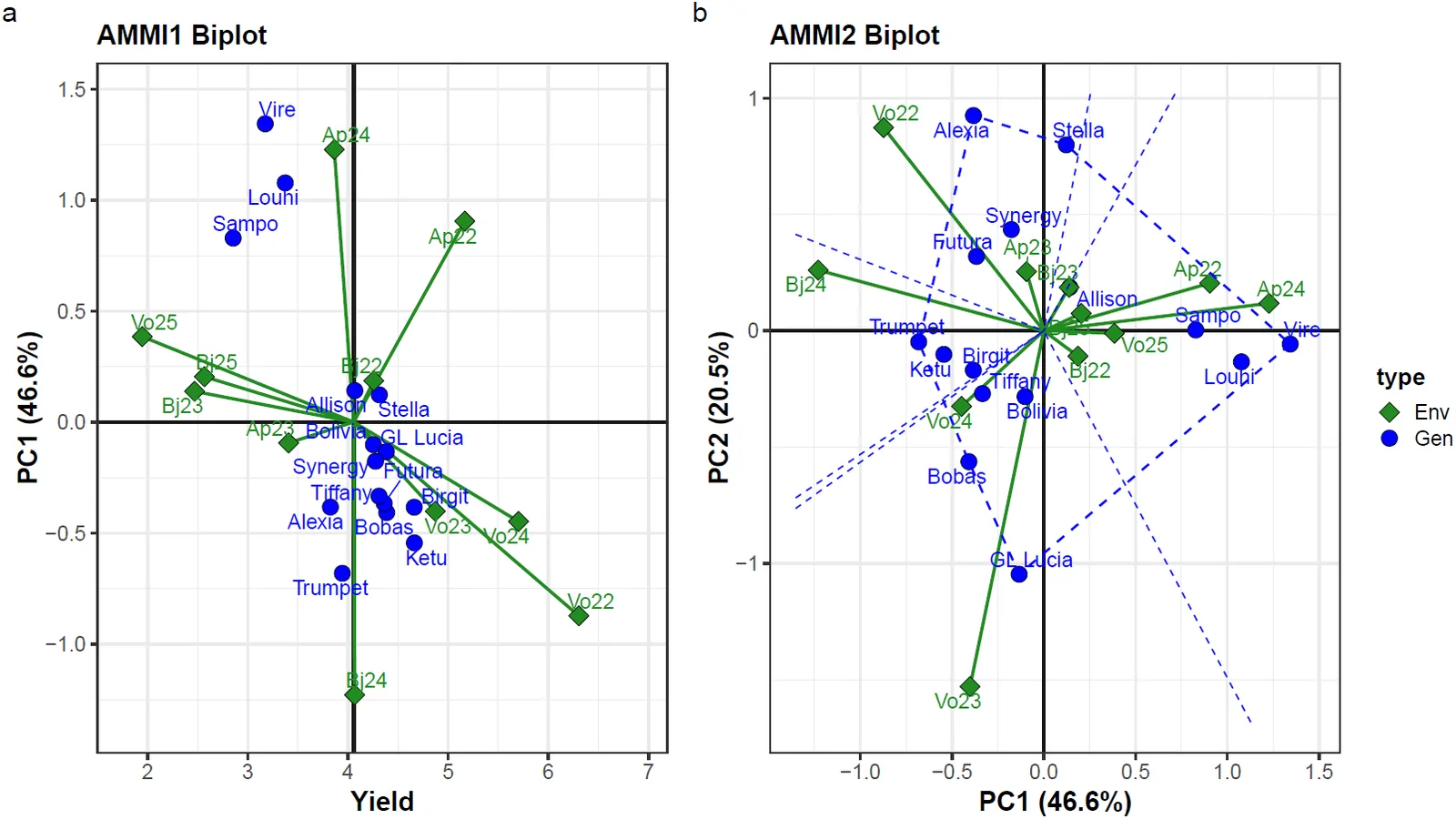
Graphical representation of genotype × environment interaction using the selected AMMI biplots. **(a)** AMMI1 biplot showing genotype and environment mean grain yield plotted against the first interaction principal component axis (IPCA1). Genotypes or environments located close to the horizontal axis exhibit relatively small interaction effects, whereas large positive or negative IPCA1 scores indicate stronger specific adaptation. **(b)** AMMI2 biplot displaying genotype and environment scores on the first two interaction principal component axes (IPCA1 and IPCA2), which together explained 67.1% of the genotype × environment interaction. Dashed lines delimit sectors of the interaction space defined by vertex genotypes, indicating groups of environments with similar patterns of genotype response.

The AMMI1 and AMMI2 biplots provided complementary graphical representations of genotype × environment interaction patterns (**Figure 1**). IPCA1 explained 46.6% of the GEI variation, while the addition of IPCA2 increased this proportion to 67.1%. The AMMI2 biplot (**Figure 1b**) displayed genotype and environment scores on the first two interaction principal component axes. Genotypes or environments located close together exhibited similar interaction patterns. Genotype-environment pairs that were close (e.g., Vire and Ap24) displayed positive GEI that increased as a function of their distance from the origin. While this biplot served primarily to visualize the structure of the GEI effects, the AMMI1 biplot (**Figure 1a**) combined genotype mean yield with IPCA1 scores, allowing the simultaneous assessment of mean yield and GEI explained by IPC1. For example, the genotypes GL Lucia and Stella had moderate mean yield along with high adaptability and yield stability across environments due to low IPCA1 score; Ketu and Birgit had highest mean yield and moderately high adaptability and yield stability; and Vire had low mean yield along with high specific adaptation to two environments of Apelsvoll (Ap22 and Ap24) out of three.

#### 3.1.3 AMMI-modelled adaptive responses and genotype recommendation

**Figure 2:**
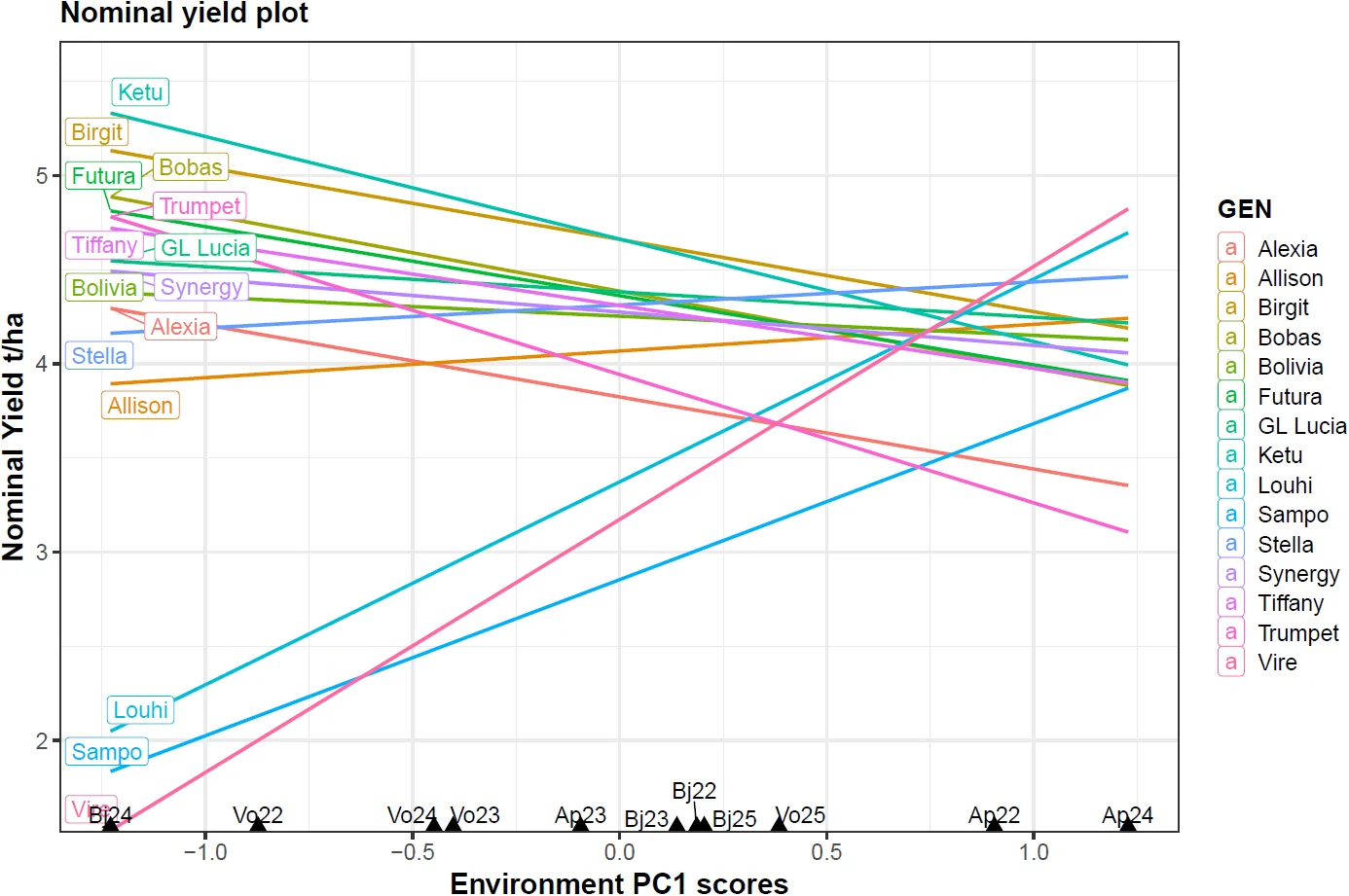
Nominal yield responses of the fifteen faba bean genotypes across the environmental gradient represented by IPCA1. Nominal yields were calculated from the AMMI1 model and plotted for the observed range of environmental IPCA1 scores. Black triangles indicate the position of the eleven trial environments along the environmental gradient. The figure illustrates contrasting adaptation patterns among genotypes and provides the basis for delineating practical mega-environments and genotype recommendations.

**Figure 2** reports the nominal yield responses of genotypes as a function of the environment ordination on IPCA1. This graph provided a simple description of cultivar adaptation that could provide a first basis for genotype recommendation. It highlighted only two mega-environments. The smaller one included just two environments of Apelsvoll (Ap22 and Ap24), in which the genotypes Vire and Louhi were top-yielding. The larger one included the remaining environments, in which the genotypes Ketu and Birgit were top-yielding. This latter mega-environment included also the third environment of Apelsvoll (Ap23). A limitation of this representation of adaptation patterns was the neglection of the GEI effects relative to IPCA2 and IPCA3. When considering them (as reported in **Table 2** for the three top-yielding genotypes in each environment), the genotype Birgit emerged as particularly suitable for the site of Bjørke, in which it was the top-ranking cultivar in three years (Bj22, Bj23 and Bj25) and the second ranking genotype in the remaining year (Bj24). The genotype Stella emerged as an additional well-performing cultivar besides Vire and Louhi in Apelsvoll based on data of two years, with genotype targeting for this site complicated by its completely different set of top-yielding genotypes in another year.

**Table 2:** Predicted grain yield (t/ha) obtained from the fitted AMMI3 model for genotypes retained in the recommendation model.

Environment	Ketu	Birgit	Futura	Synergy	Tiffany	GL Lucia	Bobas	Alexia	Stella	Vire	Louhi
Ap 22	5.27	5.37	5.22	5.33	5.07	5.15	5.00	4.75	**5.71**	*5.49*	5.43
Ap 23	3.81	**4.26**	3.57	3.52	3.43	3.58	*3.86*	*3.97*	3.71	2.27	2.61
Ap 24	4.26	3.46	4.29	4.39	4.07	3.69	3.17	2.23	*4.65*	**4.86**	*4.45*
Bj22	*4.74*	**4.84**	4.43	4.37	4.45	*4.69*	4.60	3.92	4.43	3.62	3.79
Bj23	2.73	**3.26**	2.50	2.50	2.42	2.69	*2.88*	*2.91*	2.74	1.64	1.93
Bj24	**5.29**	*5.13*	*4.88*	4.59	4.64	4.30	4.78	4.61	4.37	1.51	2.03
Bj25	2.86	**3.29**	2.61	2.59	2.57	*2.88*	*2.96*	2.76	2.79	1.86	2.11
Vo22	**7.56**	6.81	*7.51*	*7.32*	6.84	5.71	6.24	6.63	7.32	4.33	4.54
Vo23	*5.89*	5.85	4.87	4.53	5.70	**6.84**	*6.19*	3.30	3.88	3.55	3.96
Vo24	**6.95**	6.14	*6.48*	6.22	*6.50*	6.27	6.05	4.54	5.87	4.41	4.55
Vo25	1.94	**2.86**	1.65	1.68	1.73	2.42	*2.52*	*2.47*	1.99	1.38	1.72

Only genotypes ranking among the three highest-yielding in at least one environment are reported. Environments are grouped by testing location to facilitate interpretation of adaptation patterns across years. Within each environment, the highest predicted yield (based on AMMI3 model) is shown in bold and the second and third ranked genotypes are shown in italics.

The nominal yield responses in **Figure 2**, when considered with respect to all genotypes rather than just the top-yielding ones, have important implications also for breeding strategies. Breeding for wide adaptation to Norwegian environments is supported by the moderately high consistency of cultivar responses across individual environments of two locations (Vollebekk and Bjørke) and one environment out of three of the third location (Ap23) of Apelsvoll. The inconsistency for environment ordination along IPCA1 of the three environments of Apelsvoll (**Figure 2**) discourages to consider the area of this site as a sub-region object of specific breeding.

The results in **Figure 2** have implications also for the choice of genetic resources for future breeding work. They showed a relationship between adaptation pattern and geographical origin of the genotypes. The three genotypes that improved drastically their adaptive responses in the environments Ap22 and Ap24 of Apelsvoll (Vire, Louhi, and Sampo; **Figure 2**) were those of Finnish origin, the only ones displaying an early phenology and a small seed (**Supplementary Table 1, (S1)**). The widely adapted genotypes Birgit and Ketu were bred in Germany and France, respectively, and had late phenology and intermediate seed size (**Supplementary Table 1**).

### 3.2 Comparison of AMMI with mixed-model prediction

The predictive performance of the selected AMMI model was compared with the BLUP and GBLUP (calculated with the inclusion of the genomic relationship matrix between genotypes estimated from SNP data) models using the same cross-validation procedure. Although both mixed-model approaches achieved slightly lower RMSPD values than the selected AMMI3 model, the differences were small (**Supplementary Table 3, (S5)**). In particular, GBLUP showed only a marginal improvement over the BLUP model, indicating that the available marker information contributed little additional predictive ability for grain yield in this numerically limited set of genotypes. Overall, AMMI3 provided predictive accuracy comparable to both mixed-model approaches while simultaneously offering a biologically interpretable description of genotype × environment interaction.

### 3.3 Factorial regression-modelled adaptive responses

The environmental variation among the eleven trial environments was first explored by a PCA performed on a set of climatic variables that could describe the growing season. The analysis revealed clear differences among environments in their temperature and precipitation profiles, providing a basis for identifying environmental factors underlying GEI patterns. The ordination of environments in the space of the first two PC axes indicated a prevalence of the year factor, with different locations of the same year clustering together (**Supplementary Figure 3, (S6)**). Factorial regression identified the daily maximum temperature in September as the single most important environmental covariate, explaining 21.1% of the total GEI variation (**Table 3**). The addition of daily mean temperature in September as a second covariate explained an additional 17.4% of GEI variation, leading to 38.5% the share of explained GEI variation. No other climatic variable significantly improved the model according to the forward selection procedure based on AIC.

**Table 3:** Factorial regression analysis identifying climatic variables contributing to GEI for grain yield.

Variable	Df	SS	F	P	AIC	GEI % explained	GEI % cumulative
GEI	140	2332244	6.617	<0.001			
Max September	15	492376	2.41	<0.01	1389.4	21.11	21.11
Mean September	15	405999	2.27	0.01	1368.9	17.41	38.52

Sums of squares for environmental variables were obtained by scaling the corresponding coefficient of determination (R²) to the total GEI interaction sum of squares from the AMMI analysis. The final model explained 38.5% of the total GEI variation.

**Figure 3:**
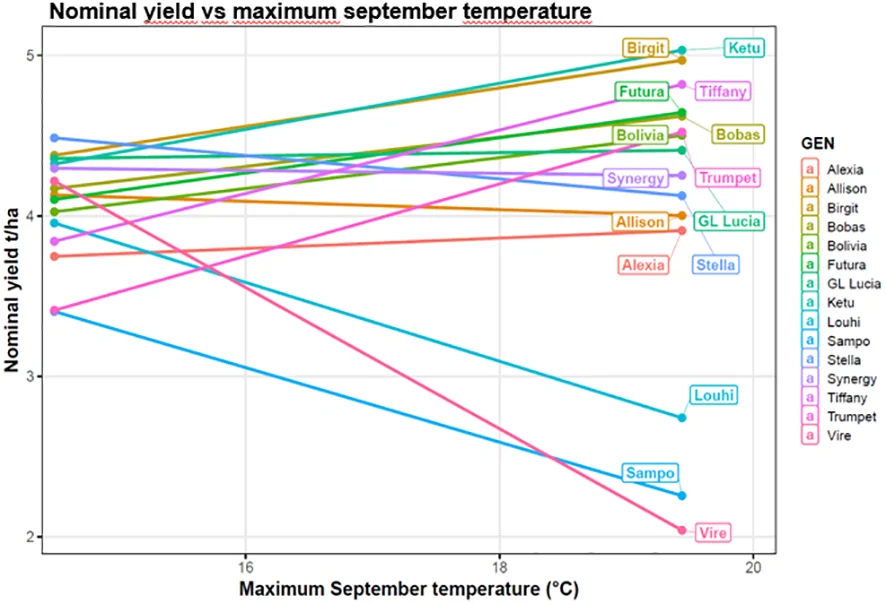
Nominal yield responses of the fifteen faba bean genotypes to maximum September temperature according to the final factorial regression model. Nominal yields were calculated over the observed range of maximum September temperatures recorded across the eleven environments. Differences in regression slopes indicate contrasting cultivar sensitivities to late-season temperature and illustrate the climatic basis of genotype × environment interaction.

The nominal yield responses derived from the factorial regression model revealed substantial differences among genotype responses to September temperature (**Figure 3**). Most genotypes, including the top-performing Ketu and Birgit, showed increasing nominal yields with increasing maximum September temperature and were predicted to be the highest yielding genotypes under warmer late-season conditions. In contrast, the early Finnish genotypes Vire, Louhi, and Sampo exhibited a marked decline of predicted yield with increasing September temperatures, indicating their specific adaptation to cooler environments. Most of the remaining genotypes displayed comparatively modest response slopes, suggesting broader adaptation across the observed temperature range.

### 3.4 Relationship between genomic and adaptive based diversity

**Figure 4:**
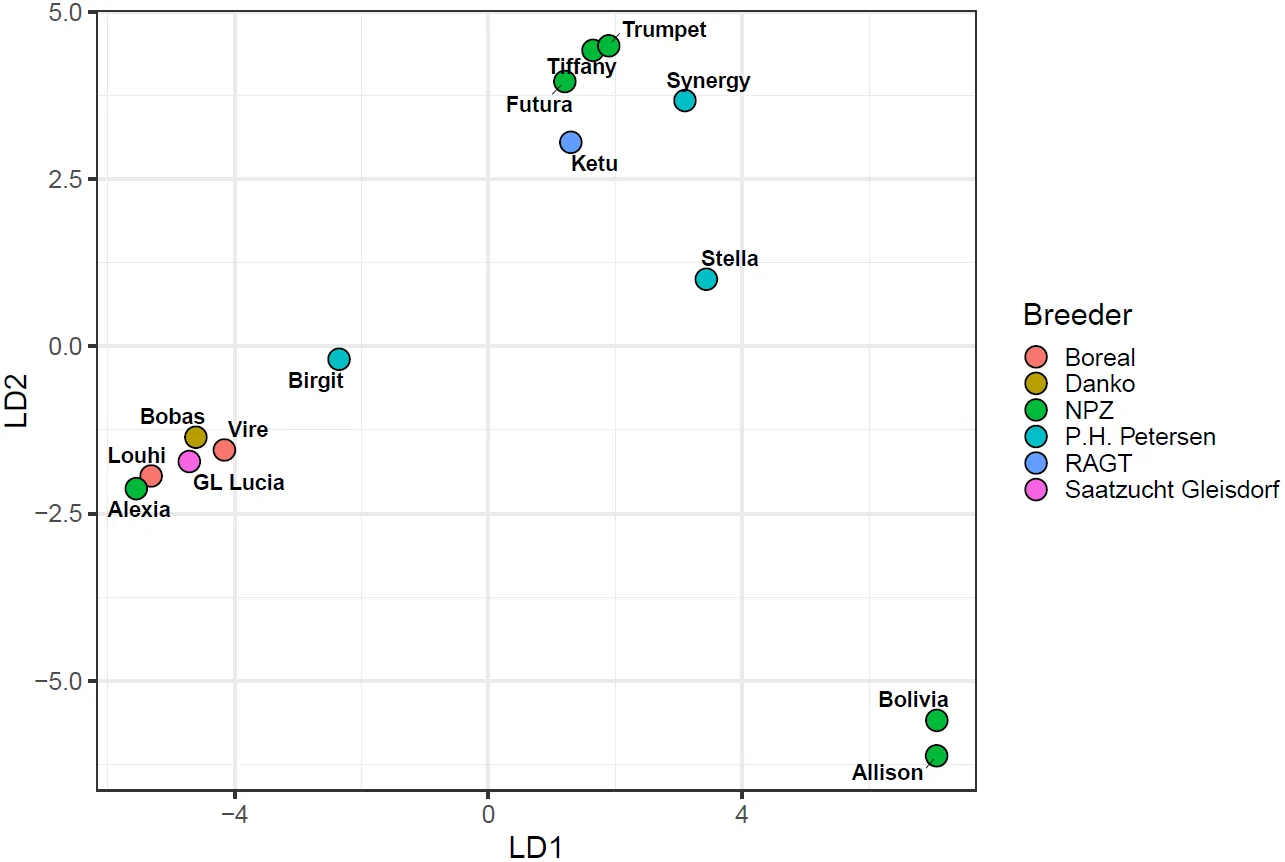
Discriminant analysis of principal components (DAPC) based on genome-wide SNP markers for the fourteen faba bean genotypes. Each point represents one genotype, and colors indicate their breeding origin.

We investigated the possible relationship between genotype adaptation patterns and the genetic makeup of the genotypes represented by ~130000 SNP markers by computing pairwise dissimilarity matrices derived from the genomic data and from adaptation patterns using Mantel’s test. A significant positive correlation was observed between the two matrices (Mantel *r* = 0.40, *P* = 0.029), indicating a moderate correspondence between molecular diversity and adaptive dissimilarity among genotypes. To provide a graphical representation of the genomic relationships among genotypes, a discriminant analysis of principal components (DAPC) based on genome-wide SNP markers is presented in **Figure 4**. These analyses revealed a clear but simple genetic structure among the genotypes that is relatively consistent with their breeding origins. In particular, the genotypes Vire and Louhi of Finnish origin were distinguished from most genotypes of German origin. Birgit, the best-performing genotype in environments positioned in the middle of **Figure 2**, was genetically distinct from both the Finnish genotypes and the genotype Ketu that was the best-performing in environments with high September temperatures.

## 4 Discussion

This study indicated the occurrence of substantial GEI for faba bean yield in Nordic environments. A similar result had previously emerged for faba bean across contrasting environments of other regions (Annicchiarico and Iannucci, 2008; Gela et al., 2023; Papastylianou et al., 2021; Skovbjerg et al., 2023; Temesgen et al., 2015), which indicated the importance of phenology and tolerance to biotic and abiotic stresses for cultivar adaptation to specific environments. While terminal drought represents a key factor for faba bean adaptation to specific environments of central and southern Europe, this study highlights the key importance of cool temperatures during late summer, which may limit the maturation of late genotypes, as a key factor that influences the adaptation to specific Nordic environments. Resistance to biotic stresses, especially *Botrytis fabae*, could also be important (Mohammadi et al., 2024). Whilst fungicide application has proven effective in controlling diseases and has become standard practice in faba bean cultivation (Grieu and Brodal, 2026), the identification of suitable genotypes for different environments in terms of optimal phenological development represents an opportunity to increase productivity.

In the AMMI analysis, the large share of GEI variation accounted for by the first GEI PC axis (46.6%) suggested that cultivar adaptation was largely driven by one major environmental factor. This agrees with the theoretical expectation that complex multi-environment datasets often contain a limited number of repeatable interaction signals embedded within substantial experimental noise (Gauch, 1992). The AMMI framework is particularly attractive because it separates these two components, allowing biologically meaningful interaction patterns to emerge while reducing the influence of random variation (Annicchiarico, 2002).

Factorial regression complemented the results of AMMI analysis by providing a physiological interpretation of the interaction structure through measured environmental variables that are directly included as causal factors of GEI effects (van Eeuwijk et al., 1996). The maximum daily temperature in September emerged as the dominant climatic factor underlying GEI, explaining approximately 21% of the total interaction variance. The importance of this variable is biologically plausible, because September largely corresponds to physiological maturity for faba bean in Norway. Temperature conditions during this period directly influence the duration of grain filling, assimilate accumulation, and maturity, particularly in late-maturing genotypes. Considering the tolerance of faba bean to episodic freezing temperatures even in late growth stage, the lower productivity of late-maturing cultivars under cool September conditions is likely associated primarily with incomplete grain filling and delayed maturity rather than direct frost damage. The contrasting slopes observed in the nominal yield response curves (**Figure 3**) are consistent with the available phenological information, indicating the importance of early-maturing genotypes such as that of Finnish material for adaptation to environments whose production is limited by the early occurrence of low temperatures during the last part of the crop cycle. However, these environments were relatively rare and occurred inconsistently even in the cooler site of Apelsvoll, highlighting the mostly superior yielding ability, adaptability and yield stability of later-maturing genotypes such as Birgit and Ketu, which were bred in central or western Europe. While a substantial proportion of GEI was captured by cultivar adaptation in response to environment variation in late-season thermal conditions, more than half of the interaction variation with biological meaning according to AMMI analysis remained unexplained by the measured climatic variables, indicating that unknown additional environmental factors contributed to cultivar performance. Rainfall, or temperature variation during earlier stages of the growing season, did not have a sizeable effect on GEI. Our results have implications also for future multi-site evaluation of foreign cultivars aimed to cultivar recommendation, suggesting, in particular, to consider Apelsvoll among the test sites because of the peculiar response of material that it may display.

The present results also have implications also for future breeding work for the region. Although the AMMI analysis suggested the presence of two possible sub-regions object of distinct breeding, various considerations support the selection of widely-adapted cultivars for Norway, namely: (i) the limited size of the smaller sub-region represented by Apelsvoll, featuring lower maximum temperatures in September; (ii) the inconsistent classification into this sub-region of the environments of this site depending on the year (with one environment out of three classified into the larger sub-region); (iii) the predicted extension of the growing season in Norway and other Nordic regions (Sjulgård et al., 2023), which could progressively decrease the frequency of environments with cool temperatures in September. Therefore, the cultivation of high yielding and moderately late genotypes might be expanded to a larger area of the agricultural area in Norway, profiting of genotypes and genetic resources that originated in more southern latitudes. Furthermore, the cultivation of faba bean might be expanded to areas which nowadays are not considered suitable because of the limited length of the growing season. Expansion into areas with a shorter growing season, currently dominated by barley, will require early maturing varieties capable of completing grain filling and reaching physiological maturity under cool autumn conditions. These areas offer considerable potential to increase national protein production. However, the multi-site selection of breeding material aimed to breeding for wide adaptation ought to include Apelsvoll among the test sites because of its peculiar GEI pattern (and/or possibly a cooler site in case of planned expansion northwards of the crop).

The integration of genomic information provided an initial indication that adaptive differentiation among genotypes had a measurable genetic basis. The moderate (r = 0.40) and significant Mantel correlation between genomic and adaptive dissimilarity indicated that genotypes sharing similar molecular backgrounds also tended to exhibit similar adaptation patterns. A few earlier studies reported a looser relationship between molecular and adaptive diversity of genotypes, with correlations of 0.26 in durum wheat (Annicchiarico et al., 2009) and 0.34 in alfalfa (Annicchiarico et al., 2020). Studies on large germplasm collection of different grain legume species assessed the relationship between molecular diversity and phenotypic diversity as expressed by a number of morphophysiological traits, reporting low correlation values (Schafleitner et al., 2015; Crosta et al., 2023; Perić et al., 2025; Annicchiarico et al., 2026) with the exception of one study in cowpea Xiong et al. (2018). However, Zanotto et al. (2025) reported a moderate correlation between molecular and phenotypic diversity in a study in which the former was limited to areas tagged for high gene density. The current correlation relative to adaptive diversity could be exploited by regional breeding programs for a preliminary screening of breeding material and novel plant introductions. Genomic predictions for yield response in specific environments, which are a major target of faba bean breeding programs (Adhikari et al., 2021), will eventually provide a more valuable tool for these purposes.

Finally, the contribution of genomic information to predictive performance was limited in the present dataset, as reflected by the very small difference between BLUP and GBLUP. This outcome is not unexpected considering the small number of genotypes available for genomic prediction. However, the observed relationship between genomic diversity and adaptive responses suggests that genomic information may become increasingly valuable when integrated with larger multi-environment breeding populations.

## 5 Conclusions

The AMMI analysis of multi-environment trials proved effective for describing genotype × environment interaction and identifying elite faba bean genotypes for Norwegian growing conditions. Factorial regression identified late-season temperature as the principal environmental driver of cultivar adaptation. Our results have valuable implications for future multi-location cultivar testing aimed at cultivar recommendations, highlighting the importance of sites with low late-season temperatures descending from the specific-adaptation effects that they may reveal. They have important implications also regional breeding programs, which could conveniently have a wide adaptation target based on material with moderately late phenology and multi-site selection across locations with contrasting GEI patterns that were concurrently identified. Finally, the observed relationship between genomic and adaptive diversity suggests that genomic information may contribute to the identification of adapted breeding material when using larger populations.

## Data Availability

The datasets presented in this study can be found in online repositories. The names of the repository/repositories and accession number(s) can be found below: https://doi.org/10.5281/zenodo.21342380, 10.5281/zenodo.21342380.

## Supplementary material

Supplementary Table 1 (S1) Overview of the spring Faba bean varieties, origin and characteristics related to maturation time and seed size.

Supplementary Figure 1 (S2) Mean daily temperature (°C) and total precipitation (mm) per month at the three locations Vollebekk (Vo), Apelsvoll (Ap) and Bjørke (Bj) in all growing seasons (2022-2025).

Supplementary Table 2 (S3) Overview of management practices applied for each field trial at the locations Apelsvoll (Ap), Bjørke (Bj), and Vollebekk (Vo) in the seasons 2022-2025.

Supplementary Figure 2 (S4) Cross-validation results of the AMMI family of models. Predictive performance was assessed using the root mean square predictive difference (RMSPD) obtained from 200 cross-validation iterations for models including zero (AMMI0) to ten interaction principal component axes (AMMI10). Lower RMSPD values indicate higher predictive accuracy. The red point indicates the AMMI3 model which was selected because of optimal balance between predictive accuracy and model parsimony.

Supplementary Table 3 (S5) Predictive performance of the AMMI family of models, BLUP and GBLUP assessed by cross-validation. Root mean square predictive difference (RMSPD), standard deviation (SD), standard error (SE), and 95% confidence interval (2.5% and 97.5% quantiles) are reported from 200 cross-validation iterations. Lower RMSPD values indicate higher predictive accuracy. The AMMI3 model (highlighted in bold) was selected for subsequent analyses because it represented the best compromise between predictive accuracy and model parsimony, whereas BLUP and GBLUP (also in bold) achieved the lowest overall prediction error.

Supplementary Figure 3 (S6) Principal component analysis (PCA) of the eleven trial environments based on monthly climatic variables recorded during the growing season, average daily mean, minimum and maximum air temperature together with total precipitation from May to September. The PCA summarizes climatic similarities among environments and was used to provide an overview of environmental variation prior to factorial regression analysis.
