# Supplementary material for: Adaptation of faba bean cultivars across Norwegian environments: identification of elite material and definition of optimal breeding and cultivar testing procedures
Source: Front Plant Sci. 2026 Sep 8;17:1936638. doi: 10.3389/fpls.2026.1936638 (PMC13595569; doi:10.3389/fpls.2026.1936638)
Supplement: Supplementary file 1 [file SupplementaryFile1.docx]

**SUPPLEMENTARY MATERIAL**

**Supplementary table 1 (S1)** Overview of the spring Faba bean varieties, origin and characteristics related to maturation time and seed size.

| Variety | Breeding company | Country | Early/late | Seed size |
| --- | --- | --- | --- | --- |
| Alexia | NPZ | Germany | Late | Equina |
| Allison | NPZ | Germany | Late | Equina |
| Birgit | P.H. Petersen | Germany | Late | Equina |
| Bobas | Danko | Poland | Late | Equina |
| Bolivia | NPZ | Germany | Late | Equina |
| Ketu | RAGT | France | Late | Equina |
| Futura | NPZ | Germany | Late | Equina |
| GL Lucia | Saatzucht Gleisdorf | Germany | Late | Equina |
| Louhi | Boreal | Finland | Early | Minor |
| Sampo | Boreal | Finland | Early | Minor |
| Stella | P.H. Petersen | Germany | Late | Equina |
| Synergy | P.H. Petersen | Germany | Late | Equina |
| Tiffany | NPZ | Germany | Late | Equina |
| Trumpet | NPZ | Germany | Late | Equina |
| Vire | Boreal | Finland | Early | Minor |

**Supplementary figure 1 (S2).** Mean daily temperature (°C) and total precipitation (mm) per month at the three locations Vollebekk (Vo), Apelsvoll (Ap) and Bjørke (Bj) in all growing seasons (2022-2025).

Mean daily Temperature °C

|  | May | June | July | August | Sptember |
| --- | --- | --- | --- | --- | --- |
| Vo22 | 10.6226 | 15.73 | 16.5065 | 16.3419 | 12.4875 |
| Bj22 | 10.1346 | 15.78 | 15.6806 | 15.5935 | 10.2233 |
| Apv22 | 9.53871 | 15.5133 | 15.4903 | 15.6323 | 10.8136 |
| Vo23 | 12.5583 | 17.7267 | 15.3065 | 15.3032 | 13.8367 |
| Bj23 | 11.95 | 17.6733 | 15.1161 | 14.6194 | 12.731 |
| Apv23 | 11.5857 | 17.29 | 14.7968 | 14.3032 | 13.0478 |
| Vo24 | 15.0226 | 14.3167 | 15.8742 | 15.429 | 14.3 |
| Bj24 | 14.9552 | 14.2 | 15.6 | 14.7806 | 10.7267 |
| Apv24 | 14.4871 | 13.6967 | 15.3387 | 14.6355 | 10.7933 |
| Vo25 | 11.15 | 14.2667 | 19.1452 | 15.2258 |  |
| Bj25 | 10.8258 | 13.7033 | 18.7387 | 14.7516 | 11.8667 |

Total Precipitation mm

|  | May | June | July | August | Sptember |
| --- | --- | --- | --- | --- | --- |
| Vo22 | 57.8 | 68.8 | 98.6 | 37.4 | 3 |
| Bj22 | 39.4231 | 105.1 | 74.5 | 77.5 | 68.5 |
| Apv22 | 27.9 | 79.9 | 58 | 77.2 | 27.4 |
| Vo23 | 5.75 | 34 | 155.6 | 189.8 | 96.8 |
| Bj23 | 8.01818 | 22.5 | 138.4 | 165.9 | 113.586 |
| Apv23 | 8 | 42.2 | 204.2 | 255.1 | 72.3 |
| Vo24 | 37.6 | 135 | 169.4 | 100.6 | 48.9667 |
| Bj24 | 22.4483 | 80.2 | 158.5 | 106.3 | 65.9 |
| Apv24 | 16.4 | 79.5 | 143.7 | 65.6 | 84.3 |
| Vo25 | 38.8 | 102.8 | 85.4 | 113.4 |  |
| Bj25 |  | 46.2 | 96.6 | 28 | 70.4 |

**Supplementary table 2 (S3).** Overview of management practices applied for each field trial at the locations Apelsvoll (Ap), Bjørke (Bj), and Vollebekk (Vo) in the seasons 2022-2025.

| Treatment | Ap2022 | Bj2022 | Vo2022 | Ap2023 | Bj2023 | Vo2023 | Ap2024 | Bj2024 | Vo2024 | Bj2025 | Vo2025 |
| --- | --- | --- | --- | --- | --- | --- | --- | --- | --- | --- | --- |
| Sowing date | 25.Apr | 06.May | 22.Apr | 11.May | 10.May | 08.May | 07.May | 03.May | 24.May | 30.Apr | 02.May |
| Harvest date | 22.Sep | 11.Okt | 08.Sep | 29.Sep | 24.Sep | 30.Sep | 23.Okt | 30.Sep | 13.Sep | 20.Aug | 03.Sep |
| Fertilizer | no | no | 30kg OPTI- PK 0-11-21 | no | no | 30kg OPTI-PK 0-11-21 | no | no | 30kg OPTI-PK 0-11-21 | no | no |
| Plot size, width*length, m | 1.5*8 | 1.5*6 | 1.5*8 | 1.5*8 | 1.5*6 | 1.6*6 | 1.5*8 | 1.5*6 | 1.6*6 | 1.5*6 | 1.6*6 |
| Herbicide | Basagran | Basagran | Basagran |  | Basagran | Basagran |  | Basagran | Basagran | Basagran | Basagran |
| Fungicide | Signum | Signum | Signum | Signum | Signum | Signum | Signum | Signum | Signum/ Pictor  Active | Signum | Signum |
| No. reps treated/untreated fungicide | 4/0 | 2/2 | 4/0 | 4/0 | 2/2 | 4/0 | 2/2 | 2/2 | 2/2 | 2/2 | 2/2 |

**Supplementary Figure 2 (S4).** Cross-validation results of the AMMI family of models. Predictive performance was assessed using the root mean square predictive difference (RMSPD) obtained from 200 cross-validation iterations for models including zero (AMMI0) to ten interaction principal component axes (AMMI10). Lower RMSPD values indicate higher predictive accuracy. The red point indicates the AMMI3 model which was selected because of optimal balance between predictive accuracy and model parsimony.

**Supplementary Table 3 (S5).** Predictive performance of the AMMI family of models, BLUP and GBLUP assessed by cross-validation. Root mean square predictive difference (RMSPD), standard deviation (SD), standard error (SE), and 95% confidence interval (2.5% and 97.5% quantiles) are reported from 200 cross-validation iterations. Lower RMSPD values indicate higher predictive accuracy. The AMMI3 model (highlighted in bold) was selected for subsequent analyses because it represented the best compromise between predictive accuracy and model parsimony, whereas BLUP and GBLUP (also in bold) achieved the lowest overall prediction error.

| **MODEL** | **mean** | **sd** | **se** | **Q2.5** | **Q97.5** |
| --- | --- | --- | --- | --- | --- |
| AMMI0 | 77.70 | 5.12 | 0.36 | 69.96 | 88.80 |
| AMMI1 | 68.55 | 3.59 | 0.25 | 62.27 | 75.82 |
| AMMI2 | 65.59 | 4.19 | 0.30 | 58.79 | 74.88 |
| **AMMI3** | 62.24 | 4.49 | 0.32 | 54.84 | 70.26 |
| AMMI8 | 61.58 | 4.52 | 0.32 | 54.44 | 70.96 |
| AMMI4 | 61.46 | 4.19 | 0.30 | 54.50 | 69.64 |
| AMMI5 | 61.26 | 4.55 | 0.32 | 53.80 | 70.03 |
| AMMI7 | 61.21 | 4.60 | 0.33 | 53.40 | 69.64 |
| AMMI9 | 61.04 | 4.27 | 0.30 | 53.70 | 69.30 |
| AMMI6 | 61.02 | 4.52 | 0.32 | 53.07 | 69.44 |
| AMMIF | 60.73 | 4.15 | 0.29 | 53.54 | 68.46 |
| **BLUP** | 60.47 | 4.62 | 0.33 | 52.43 | 69.68 |
| **GBLUP** | 60.46 | 4.61 | 0.33 | 52.47 | 69.66 |

**Supplementary figure 3 (S6).** Principal component analysis (PCA) of the eleven trial environments based on monthly climatic variables recorded during the growing season, average daily mean, minimum and maximum air temperature together with total precipitation from May to September. The PCA summarizes climatic similarities among environments and was used to provide an overview of environmental variation prior to factorial regression analysis.
